# A radical form of nitric oxide inhibits porcine circovirus type 2 replication in vitro

**DOI:** 10.1186/s12917-019-1796-x

**Published:** 2019-02-01

**Authors:** Tao Xue, Jizong Li, Chuanmin Liu

**Affiliations:** 1 School of Pharmacy, Linyi University, Linyi, 276000 China; 2 Institute of Veterinary Medicine, Jiangsu Academy of Agricultural Sciences,Key Laboratory of Veterinary Diagnosis, Jiangsu Academy of Agricultural Sciences, Key Laboratory of Veterinary Biological Engineering and Technology, Ministry of Agriculture, Nanjing, 210014 China

**Keywords:** Nitric oxide, Porcine circovirus type 2, In vitro

## Abstract

**Background:**

Porcine circovirus type 2 (PCV2) is the causal agent of postweaning multisystemic wasting syndrome (PMWS), causing large economical losses of the global swine industry. Nitric oxide (NO), as an important signaling molecule, has antiviral activity on some viruses. To date, there is little information on the role of NO during PCV2 infection.

**Results:**

We used indirect fluorescence assay (IFA), TCID_50_, real-time RT-qPCR and western blot assay to reveal the role of NO in restricting PCV2 replication. PCV2 replication was inhibited by a form of NO, NO^**•**^, whereas PCV2 was not susceptible to another form of NO, NO^+^.

**Conclusion:**

Our findings indicate that the form of NO^**•**^ has a potential role in the fight against PCV2 infection.

## Background

Porcine circovirus (PCV) is a small, non-enveloped virus with a circular, single-stranded DNA genome, belonging to the family *Circoviridae*, genus *Circovirus* [1]. Three genotypes of PCV are known to us, including PCV1, PCV2 and PCV3 [2]. PCV1 is non-pathogenic to pigs, PCV3 is characterized by PDNS, reproductive failure, as well as cardiac and multisystemic inflammation, PCV2 is pathogenic as the agent of porcine circovirus associated disease (PCVAD), which is a globally emerging disease with a huge impact on swine-producing countries [3, 4]. Post-weaning multisystemic wasting syndrome (PMWS), the first recognized PCVAD in Canada in 1991 [5], appears in growing pigs at the age of 5 to 18 weeks characterized by clinical signs of progressive weight loss, fever, enlarged lymph nodes, respiratory distress, and, occasionally, jaundice and diarrhea [6]. Common measures taken to prevent and control PCVAD were considered as follows: management improvement, control of co-infections, control of breeding and semen quality, improvement of herd nutrition, serum therapy and vaccination [7]. To our knowledge, no effective antiviral therapy has been adopted to control PCV2 infection in clinical settings.

NO is a short-lived and reactive free radical that is chemically able to diffuse within biological systems. In mammalian cells, the production of NO is catalyzed by a family of NO synthases (NOSs), which facilitate the nicotinamide adenine dinucleotide phosphate (NADPH)-dependent reaction of L-arginine with O_2_, to yield NO and L-citrulline [8]. Besides, NO-generating compounds, such as sodium nitroprusside (SNP), S-nitroso-acetylpenicillamine (SNAP), S-nitrosoglutathion (GNSO) and so on, can also release NO. It has been reported that SNP alone provides NO^+^ as the major form of NO in culture media while SNP in the presence of ascorbate generates mostly the reduced form of NO, NO^**•**^ [9]. The antiviral activity of NO activity has been demonstrated against a variety of viruses including Marek’s disease virus, astrovirus, dengue virus type 2, herpes simplex virus type 1 and Theiler’s murine encephalomyelitis virus [10–14]. Our previous study show that NO-generating compound GSNO suppresses PCV2 infection in vitro and in vivo [15], and the other studies also confirmed that PCV2 replication could be suppressed by L-arginine in vivo [15, 16]. As L-arginine is the natural precursor of NO [17], whether the inhibition of PCV2 induced by L-arginine is associated with NO, it remains unknown to us.

In this study, a radical form of NO, NO^+^ was generated from SNP alone, while another form of NO, NO^**•**^ was released from SNP plus vitamin C (VC), and the effects of exogenous NO on PCV2 replication was investigated in PK-15 cells.

## Results

### Cytotoxicity

**Fig. 1:**
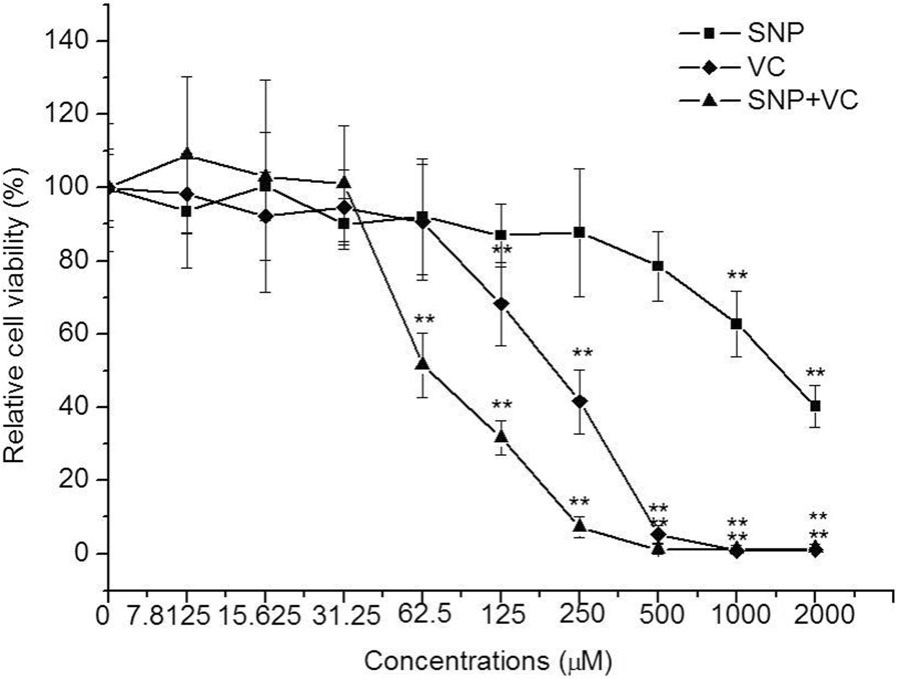
Cytotoxicity of SNP, VC and SNP plus VC on PK-15 cells tested by MTT assay. After incubation with the drugs for 72 h, MTT was added into each well. The cells were cultured for another 4 h and 200 μl DMSO was added into each well for dissolving formazan, then A570 of the samples were determined with a microplate reader (Sunrise, TECAN Co., Swiss). Relative viability was calculated according to the equation: Relative viability (%) = A570 of the drug-treated sample/A570 of the untreated sample × 100. Data shown were means ± SD from three independent experiments. Statistical significance was indicated in relation to the untreated control group: *P < 0.05, **P < 0.01

The results indicated that MNTCs of SNP (Sigma, USA), VC (Sigma, USA) and SNP plus VC to PK-15 cells were 500, 62.5 and 31.25 μM respectively (Fig. 1). Concentrations of the drugs used in this study were all within MNTCs. N-acetylpenicillamine (NAP) (Sigma, USA), lacking the S-nitroso group, does not release NO and was used as the control of NO donor. 60 μM NAP did not have cytotoxicity to PK-15 cells (data not shown).

### NO production

**Fig. 2:**
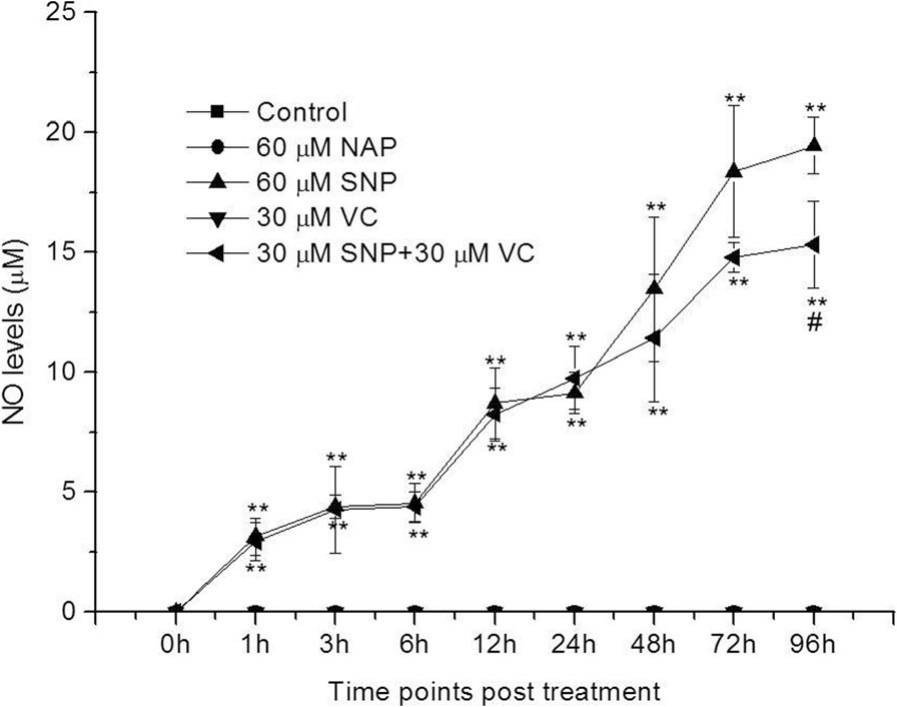
Kinetics of NO production in culture supernatant of PK-15 cells. When 80% confluent monolayers formed, NAP, SNP, VC and SNP plus VC were added into 96-well cell culture plates, four wells for each treatment, and nontreated cells served as the control. Supernatant from each sample was collected at different time points, then NO levels were quantitated by Griess reaction with a microplate reader at 540 nm and compared to a standard curve made from sodium nitrite. Data were presented as means ± SD from three independent experiments (**P < 0.01 vs the untreated control group, #*P* < 0.05 vs SNP-treated group)

**Fig. 3:**
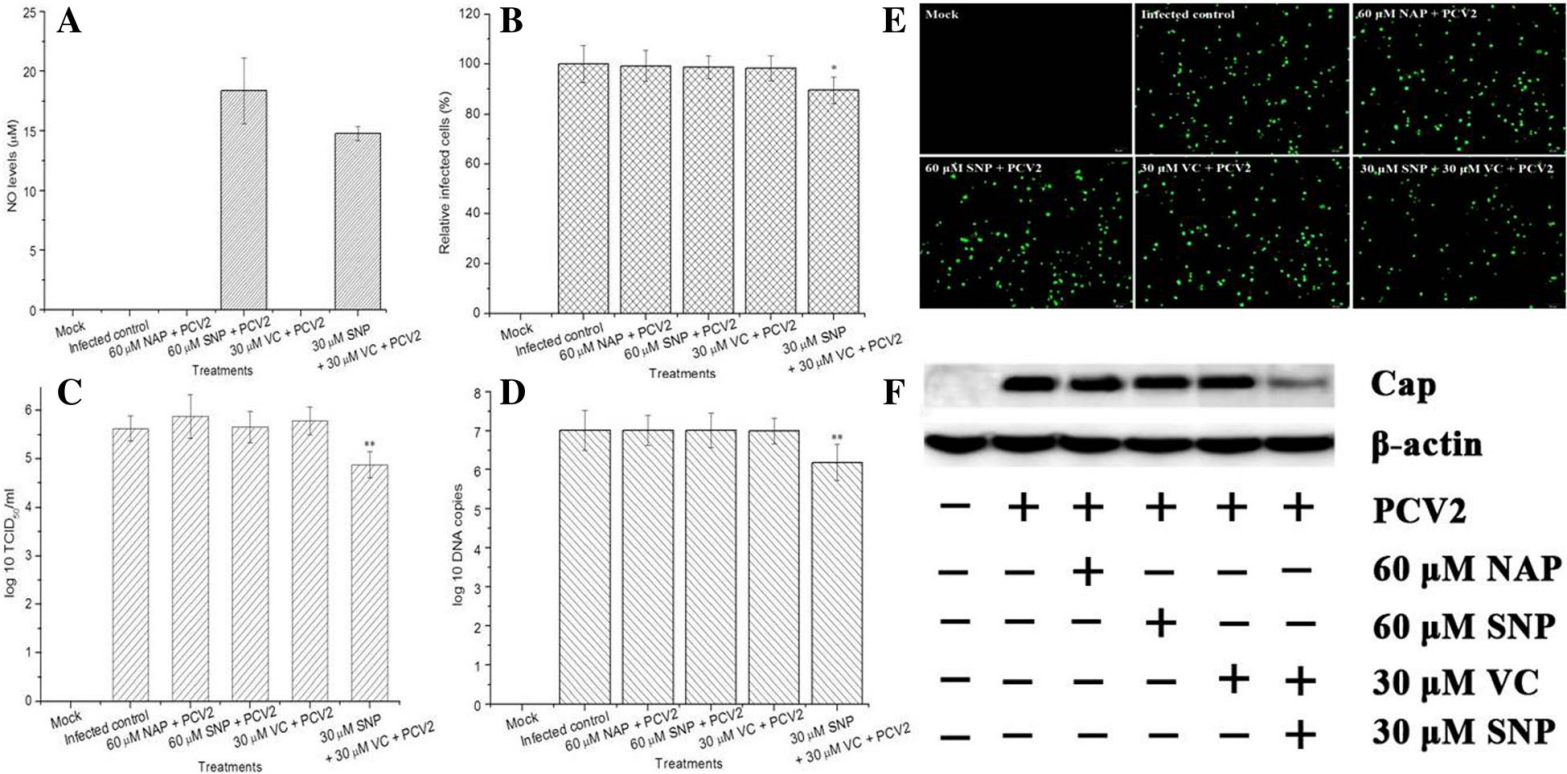
Effects of exogenous NO on PCV2 replication in PK-15 cells. When monolayers reached about 50% in each well in 24-well cell culture plates, the cells were incubated with NAP or SNP or VC or SNP plus VC for 6 h, four wells for each treatment. After washing, the cells were infected with PCV2 (1 MOI), and then cultured with the drugs for another 72 h. Nontreated cells served as mock, and the infected-cells without drug treatment were infected control. As for IFA (**a**), the cells in different groups were washed with PBS, then fixed with cold methanol, and all stained for PCV2. Since FITC-Fluorescence could be observed in PCV2-infected cells, the appearance of PCV2-positive cells was judged by FITC staining intensity, examined under a fluorescence microscope (Olympus, Japan). In addition, supernatant from each well was collected for determination of NO production (**b**), meanwhile, the cells in each sample were also gathered for assay of relative infected cells (**c**), virus titers (**d**), virus DNA copies (**e**) and virus Cap expression. NO levels were quantitated by Griess reaction. Relative infected cells were determined by flow cytometry, calculated as a percentage of infected control. Virus titers were detected by IFA, evaluated by Reed-Muench method. Virus DNA copies were measured by qPCR. virus Cap expression was determined by western blot assay. Data were presented as means ± SD from three independent experiments (*P < 0.05, **P < 0.01 vs infected control)

Kinetics of NO demonstrated that either 60 μM SNP or 30 μM SNP plus 30 μM VC generated similar amount of NO in supernatant of PK-15 cells at 0—72 h (*P* > 0.05) (Fig. 2). In addition, there was no significant difference in NO levels between 60 μM SNP-treated group and 30 μM SNP plus 30 μM VC-treated group at 72 h post PCV2 infection (*P* > 0.05) (Fig. 3 a).

### Antiviral activities

As presented in Fig. 3e, PCV2-positive cells decreased markedly in the presence of SNP plus VC when compared to those in infected control, whereas almost no visible alteration was shown in the group treated with SNP alone. Inhibitory effects of NO on PCV2 replication were further evaluated by relative infected cells, virus titers, PCV2 DNA copies and PCV2 Cap expression. As shown in Fig. 3, treatment with SNP plus VC led to significant increase in relative infected cells (Fig. 3e), virus titers (Fig. 3c), PCV2 DNA copies (Fig. 3d) and PCV2 Cap expression (Fig. 3f) in relation to infected control (*P* < 0.05 or *P* < 0.01). However, the above-mentioned parameters showed no significant difference between SNP-treated group and infected control group (*P* > 0.05). Our data suggested that NO^**•**^ generated from SNP plus VC played a critical role in blocking PCV2 replication, while NO^+^ released from SNP alone did not exert antiviral activity during PCV2 infection.

## Discussion

Some events involved in PCV2 replication have been defined by previous studies. Wei (2008) reported that NF-κB activation is important for PCV2 replication [18]. Another report revealed that PCV2 deployed PKR-like endoplasmic reticulum kinase (PERK) and glucose-regulated protein 78 (GRP78) for its enhanced replication in PK-15 cells [19]. A recent study found that G0/G1 cell cycle arrest induced by PCV2 may provide favorable conditions for viral protein expression and progeny production [20]. Meanwhile, enhancement of PCV2 replication induced by LPS, IFN-α/IFN-γ and concanavalin A was demonstrated [21–23], whereas decreased PCV2 replication mediated by L-arginine, matrine and selenium was confirmed in vitro and/or in vivo [15, 24, 25]. Although the antiviral activity of NO has been proved by much research work, regulation of PCV2 replication induced by NO remains unknown to us. In this study, treatment of infected cells with NO^**•**^ generated from SNP plus VC resulted in suppression of the growth of PCV2 while treatment with NO^+^ generated from SNP alone had no effect. Our data implied that the inhibitory effect of NO on PCV2 replication was induced by the radical form of NO^**•**^, not NO^+^. Interestingly, A previous report revealed that NO^**•**^ released from SNP plus ascorbate could delay and block rabies replication in neuroblastoma cells, while viral replication was not influenced by NO^+^ generated from SNP alone, and this data was similar to our study [26]. The mechanisms involved in the antiviral properties of NO were partially confirmed in previous studies, indicating that NO played an important role in regulation of viral protease [27], innate immunity of the host [11], as well as viral protein and nucleic acid synthesis [28]. For PCV2, we found that NO generated from SNP plus VC inhibited synthesis of infectious virions by reducing the number of viral infected cells and by downregulation of viral titers and viral DNA copies. Reduction in the number of viral infected cells may be due to the ability of NO to inhibit cytoplasmic penetration by the virus or to suppression of transcriptional steps. However, binding, entry and transcription characteristics of PCV2 influenced by NO was not investigated here. Hence, the underlying mechanisms involved in PCV2 replication induced by NO remain to be discovered in our further study.

## Conclusions

Taken together, the radical form of NO^**•**^ generated from SNP plus VC effectively inhibits PCV2 replication in vitro, while PCV2 is not sensitive to the form of NO^**+**^ generated from SNP alone. Moreover, inhibitory effects of NO^**•**^ are directly reflected by downregulation of the number of viral infected cells, viral titers, viral DNA copies and viral Cap expression. These results provide a new potential antiviral therapy against PCV2 infection for pig industry.

## Methods

### Cell and virus

PK-15 free of PCV, purchased from the China Institute of Veterinary Drug Control (Beijing, China), were grown at 37 °C in an atmosphere of 5% CO_2_ in Dulbecco’s modified Eagle’s medium (DMEM) (Sigma, USA) supplemented with 10% fetal bovine serum (FBS) (GIBCO, USA) and 1% penicillin-streptomycin antibiotics (Sangon, China). The PCV2-Haian strain (GenBank accession number FJ712216.1) was maintained by Institute of Veterinary Medicine, Jiangsu Academy Agricultural Sciences. PCV2 was propagated in PK-15 cells, harvested after 72 h incubation, and stored at − 70 °C until use.

### Experimental design

#### Cytotoxicity assay

To exclude the possibility that the detected antiviral effect of NO on PCV2 replication might have resulted from toxicity to the cells, MTT method was used to determine the maximum non-cytotoxic concentration (MNTC) of the exogenous NO donors according to a previous study [24]. In brief, PK-15 cells were seeded in 96-well plates 24 h prior and grown to 80% confluence, then treated with different concentrations of the drugs as serial two-fold dilutions, with eight wells for each concentration. After incubation for 72 h, the viability of PK-15 cells were evaluated with a colorimetric MTT assay. The absorbance at 570 nm (A570) of each well was measured with a microliter enzyme-linked immunosorbent assay reader (Sunrise, TECAN Co., Switzerland).

#### Measurement of NO

NO production was assessed by a colorimetric assay using the Griess reaction [29]. Briefly, at various time points during cell culture, the supernatants (100 μl/well) were harvested, and incubated with an equal volume of Griess solution (1% sulfanilamide, and 0.1% naphthyl ethylene diamine dihydrochloride in 5% phosphoric acid) (Sigma, USA) for 10 min at room temperature. The absorbance was read at 540 nm, and the concentrations of NO were determined from a least squares linear regression analysis of a standard curve for sodium nitrite.

#### Antiviral assay

To investigate the antiviral effects of NO during PCV2 infection, when 80% confluent PK-15 cells formed in 24-well cell culture plates, 60 μM NAP, 60 μM SNP, 30 μM VC and 30 μM SNP plus 30 μM VC were added into each well respectively. After 6 h incubation, the drug-treated groups were inoculated with PCV2 (1 MOI, Haian strain, GenBank accession number FJ712216.1) for additional 72 h. Furthermore, the untreated cells, cultured in medium alone, was considered as the mock group, while the group infected with PCV2 (1 MOI) alone was used as infected control. The antiviral activity of NO was determined by the appearance of PCV2-infected cells, virus titers and PCV2 DNA copies.

#### TaqMan-based real-time PCR

The viral DNA was extracted by using the Colume Viral DNAout Kit (TIANDZ, Beijing, China) according to the manufacturer’s instructions. The total DNA was stored at − 70 °C until use. PCV2 DNA copies was determined by TaqMan-based qPCR. The primers and TaqMan probe specific for PCV2 were designed as follows: forward primer 5’-TAAATCTCATCATG TCCACATTCCA-3′, reverse primer 5’-CGTTACCGCTGGAGAAGGAA-3′ and TaqMan probe 5′-[6-FAM] AATGGCATCTTCAACACCCGCCTCT [TAMRA]-3′. A recombinant pGEM-T easy vector (TaKaRa, China) containing PCV2 genome insert was constructed by us and the qPCR standard curve was generated by analysis of tenfold serial dilutions ranging from 10^2^ to 10^7^ copies. Each 25 μl qPCR reaction was run in triplicate containing 12.5 μl Premix Ex Taq (2×), 0.5 μL of each primer (10 μM), 0.5 μl TaqMan probe (10 μM), 0.5 μl ROX Reference DyeII (50×), 1 μl of DNA template, and 9.5 μl of deionized water. qPCR was performed on 7500 Real-Time PCR Systems (Applied Biosystems, USA) by using the following thermal cycles: 95 °C for 30 s, 40 cycles at 95 °C for 5 s, and 60 °C for 34 s.

#### Indirect fluorescence assay (IFA)

PCV2-positive cells and virus titers were observed by indirect fluorescence assay (IFA) as described previously [30]. Briefly, cells were fixed with acetone for 10–15 min at 4 °C. Fixed cells were washed with PBS, and then incubated with porcine anti-PCV2 antibody at 37 °C for 1 h. After washing three times with PBS, cells were incubated with a fluorescein isothiocyanate (FITC)-labeled rabbit anti-pig IgG (Abcam, UK) at 37 °C for 45 min. The cells positive for PCV2 viral antigens were counted in six field of view using a fluorescence microscope (Olympus, IX51, Japan). The virus titers were calculated by Reed-Muench method [31].

#### Flow cytometry

The percentage of PCV2-infected cells was analyzed by flow cytometry according to a previous study [32]. Briefly, cells were harvested and fixed with methanol 10–15 min at 4 °C and incubated with PCV2 antiserum at 37 °C for 1 h, then incubated with the FITC-labeled rabbit anti-pig IgG at 37 °C for 45 min. Stained cells were analyzed with a FACSAria flow cytometer (BD Biosciences, USA) (Dvorak et al., 2013).

#### Western blot analysis

PCV2 capsid protein (Cap) was analyzed by western blot assay. The PK-15 cells were harvested, and the protein was separated on 12% SDS-PAGE gels and transferred onto nitrocellulose membranes. Non-specific binding was blocked with 1% BSA. The membrane was probed with monoclonal antibodies (mAbs) against the Cap or mouse anti-β-actin monoclonal (BIOSS, Beijing, China). The membrane was probed with a goat anti-mouse IgG antibody conjugated to horseradish peroxidase (HRP) (BIOSS, Beijing, China). Protein was detected using enhanced chemiluminescence (ECL) reagents (Vazyme, Nanjing, China).

#### Statistical analysis

Tests of significance were performed using Duncan’s multiple-range test after one-way ANOVA by SPSS 17.0 statistics software. Data were presented as means ± SD. A *P*-value less than 0.05 was considered statistically significant.

## Abbreviations

Cap: Capsid protein
ECL: Enhanced chemiluminescence
GNSO: S-nitrosoglutathion
HRP: Horseradish peroxidase
IFA: Indirect fluorescence assay
mAbs: Monoclonal antibodies
MNTC: Maximum non-cytotoxic concentration
NAP: N-acetylpenicillamine
NO: Nitric oxide
NOSs: NO synthases
PCV2: Porcine circovirus type 2
PCVAD: Porcine circovirus associated disease
PMWS: Postweaning multisystemic wasting syndrome
SNAP: S-nitroso-acetylpenicillamine
SNP: Sodium nitroprusside
VC: Vitamin C

## References

[1] Tischer I, Gelderblom H, Vettermann W, Koch MA (1982). A very small porcine virus with circular single-stranded DNA. Nature.

[2] Palinski R, Pineyro P, Shang P, Yuan F, Guo R, Fang Y, Byers E, Hause BM. A novel porcine circovirus distantly related to known circoviruses is associated with porcine dermatitis and nephropathy syndrome and reproductive failure. J Virol. 2017;91(1):e01879–16. 10.1128/JVI.01879-16 PMC5165205 27795441

[3] Meng XJ (2012). Spread like a wildfire--the omnipresence of porcine circovirus type 2 (PCV2) and its ever-expanding association with diseases in pigs. Virus Res.

[4] Ha Z, Xie CZ, Li JF, Wen SB, Zhang KL, Nan FL, Zhang H, Guo YC, Wang W, Lu HJ (2018). Molecular detection and genomic characterization of porcine circovirus 3 in pigs from Northeast China. BMC Vet Res.

[5] Meehan BM, McNeilly F, Todd D, Kennedy S, Jewhurst VA, Ellis JA, Hassard LE, Clark EG, Haines DM, Allan GM (1998). Characterization of novel circovirus DNAs associated with wasting syndromes in pigs. J Gen Virol.

[6] Darwich L, Segales J, Mateu E (2004). Pathogenesis of postweaning multisystemic wasting syndrome caused by porcine circovirus 2: an immune riddle. Arch Virol.

[7] Grau-Roma L, Fraile L, Segales J (2011). Recent advances in the epidemiology, diagnosis and control of diseases caused by porcine circovirus type 2. Vet J.

[8] Treuer AV, Gonzalez DR (2015). Nitric oxide synthases, S-nitrosylation and cardiovascular health: from molecular mechanisms to therapeutic opportunities (review). Mol Med Rep.

[9] Murphy ME, Sies H (1991). Reversible conversion of nitroxyl anion to nitric oxide by superoxide dismutase. Proc Natl Acad Sci U S A.

[10] Xing Z, Schat KA (2000). Inhibitory effects of nitric oxide and gamma interferon on in vitro and in vivo replication of Marek's disease virus. J Virol.

[11] Koci MD, Kelley LA, Larsen D, Schultz-Cherry S (2004). Astrovirus-induced synthesis of nitric oxide contributes to virus control during infection. J Virol.

[12] Fagundes CT, Costa VV, Cisalpino D, Amaral FA, Souza PR, Souza RS, Ryffel B, Vieira LQ, Silva TA, Atrasheuskaya A (2011). IFN-gamma production depends on IL-12 and IL-18 combined action and mediates host resistance to dengue virus infection in a nitric oxide-dependent manner. PLoS Negl Trop Dis.

[13] Mehta DR, Ashkar AA, Mossman KL (2012). The nitric oxide pathway provides innate antiviral protection in conjunction with the type I interferon pathway in fibroblasts. PLoS One.

[14] Moore TC, Bush KL, Cody L, Brown DM, Petro TM (2012). Control of early Theiler's murine encephalomyelitis virus replication in macrophages by interleukin-6 occurs in conjunction with STAT1 activation and nitric oxide production. J Virol.

[15] Liu C, Wen L, Xiao Q, He K (2017). Nitric oxide-generating compound GSNO suppresses porcine circovirus type 2 infection in vitro and in vivo. BMC Vet Res.

[16] Ren W, Yin Y, Liu G, Yu X, Li Y, Yang G, Li T, Wu G (2012). Effect of dietary arginine supplementation on reproductive performance of mice with porcine circovirus type 2 infection. Amino Acids.

[17] Baecker N, Boese A, Schoenau E, Gerzer R, Heer M (2005). L-arginine, the natural precursor of NO, is not effective for preventing bone loss in postmenopausal women. J Bone Miner Res.

[18] Wei L, Kwang J, Wang J, Shi L, Yang B, Li Y, Liu J (2008). Porcine circovirus type 2 induces the activation of nuclear factor kappa B by IkappaBalpha degradation. Virology.

[19] Zhou Y, Qi B, Gu Y, Xu F, Du H, Li X, Fang W. Porcine circovirus 2 deploys PERK pathway and GRP78 for its enhanced replication in PK-15 cells. Viruses. 2016;8:56. 10.3390/v8020056 PMC4776210 26907328

[20] Quan R, Wei L, Zhu S, Wang J, Cao Y, Xue C, Yan X, Liu J (2016). Involvement of miR-15a in G0/G1 phase cell cycle arrest induced by porcine circovirus type 2 replication. Sci Rep.

[21] Chang HW, Pang VF, Chen LJ, Chia MY, Tsai YC, Jeng CR (2006). Bacterial lipopolysaccharide induces porcine circovirus type 2 replication in swine alveolar macrophages. Vet Microbiol.

[22] Ramamoorthy S, Huang FF, Huang YW, Meng XJ (2009). Interferon-mediated enhancement of in vitro replication of porcine circovirus type 2 is influenced by an interferon-stimulated response element in the PCV2 genome. Virus Res.

[23] Lefebvre DJ, Meerts P, Costers S, Misinzo G, Barbe F, Van Reeth K, Nauwynck HJ (2008). Increased porcine circovirus type 2 replication in porcine leukocytes in vitro and in vivo by concanavalin a stimulation. Vet Microbiol.

[24] Sun N, Sun P, Lv H, Sun Y, Guo J, Wang Z, Luo T, Wang S, Li H (2016). Matrine displayed antiviral activity in porcine alveolar macrophages co-infected by porcine reproductive and respiratory syndrome virus and porcine circovirus type 2. Sci Rep.

[25] Chen X, Ren F, Hesketh J, Shi X, Li J, Gan F, Huang K (2012). Selenium blocks porcine circovirus type 2 replication promotion induced by oxidative stress by improving GPx1 expression. Free Radic Biol Med.

[26] Ubol S, Hiriote W, Anuntagool N, Utaisincharoen P (2001). A radical form of nitric oxide suppresses RNA synthesis of rabies virus. Virus Res.

[27] Saura M, Zaragoza C, McMillan A, Quick RA, Hohenadl C, Lowenstein JM, Lowenstein CJ (1999). An antiviral mechanism of nitric oxide: inhibition of a viral protease. Immunity.

[28] Akerstrom S, Gunalan V, Keng CT, Tan YJ, Mirazimi A (2009). Dual effect of nitric oxide on SARS-CoV replication: viral RNA production and palmitoylation of the S protein are affected. Virology.

[29] Legorreta-Herrera M, Rivas-Contreras S, Ventura-Gallegos J, Zentella-Dehesa A (2011). Nitric oxide is involved in the upregulation of IFN-Î^3^ and IL-10 mRNA expression by CD8â° T cells during the blood stages of P. Chabaudi AS infection in CBA/ca mice. Int J Biol Sci.

[30] Xujie L, Xiaobo W, Yi S, Jing F, Song G, Xiufan L (2011). A candidate inactivated chimeric vaccine PCV1-2 constructed based on PCV1 and PCV2 isolates originating in China and its evaluation in conventional pigs in regard to protective efficacy against PCV2 infection. Microbiol Immunol.

[31] Zhu B, Xu F, Li J, Shuai J, Li X, Fang W (2012). Porcine circovirus type 2 explores the autophagic machinery for replication in PK-15 cells. Virus Res.

[32] Dvorak CM, Puvanendiran S, Murtaugh MP (2013). Cellular pathogenesis of porcine circovirus type 2 infection. Virus Res.

